# The Usability of Continuous Monitoring Devices With Deterioration Alerting Systems in Noncritical Care Units: Scoping Review

**DOI:** 10.2196/75713

**Published:** 2026-02-10

**Authors:** Jo-Fan Pan, Dawn Dowding, David Wong, Ashley Scott, Qimeng Zhao

**Affiliations:** 1 University of Manchester, Jean McFarlane Building, Oxford Road, Manchester, M13 9PL, United Kingdom, 886 0924027585; 2 University of Leeds, Leeds, United Kingdom

**Keywords:** continuous monitoring, monitoring, wearable devices, early warning score, vital sign, alerts, alarms, deterioration

## Abstract

**Background:**

Delayed recognition of patient deterioration in a non–intensive care unit (ICU) setting contributes to serious adverse events. Continuous monitoring devices with alerting systems offer real-time data to support early detection, but their effectiveness depends on usability. While prior reviews focus on clinical outcomes, usability—defined by effectiveness, efficiency, and satisfaction—remains underexplored.

**Objective:**

This study aims to scope the evidence related to the usability of continuous monitoring devices with deterioration alerting in noncritical adult care units.

**Methods:**

A scoping review was conducted following the Joanna Briggs Institute methodology and reported in accordance with the PRISMA-ScR (Preferred Reporting Items for Systematic Reviews and Meta-Analyses extension for Scoping Reviews) guidelines. A comprehensive search of MEDLINE, Embase, Emcare, Web of Science, and IEEE Xplore was performed for studies published up to November 2024. Title and abstract screening, full-text review, and data extraction were independently conducted by 2 reviewers. Studies were included if they (1) evaluated the usability—defined as effectiveness, efficiency, or satisfaction—of continuous monitoring devices; (2) focused on adult patients in non-ICU hospital settings; (3) used primary data; (4) were published in English; and (5) described how clinicians received alerts.

**Results:**

The search identified 1284 papers, with 35 included. Most studies focused on postoperative patients in surgical wards, mainly from the United States and the Netherlands. Only 2 studies used mixed methods, and 10 reported clinician characteristics. While effectiveness (71%) and efficiency (74%) were widely studied, satisfaction (46%) and usability barriers (29%) received less attention.

**Conclusions:**

Continuous monitoring devices with deterioration alerts may reduce rapid response team calls and ICU transfers, save time, and maintain acceptable alarm frequencies with high user satisfaction. However, usability challenges persist, including technical issues, alarm fatigue, patient discomfort, and limited training or workflow integration. This review mapped current use, usability, and barriers, categorized key usability factors for improvement, and identified the need for further research on clinician perspectives and broader health care settings to enhance generalizability.

## Introduction

In 2022‐2023, the United Kingdom recorded 16.4 million hospital admissions, compared to 33.7 million in the United States and 12.1 million in Australia [1-3]. Many of these patients may experience severe adverse events (SAEs), such as cardiac arrests or intensive care unit (ICU) admissions, during their hospital stay, with estimates ranging from 5% to 10% [4,5]. According to UK data, 23% of in-hospital SAEs resulting in sudden death are due to failures to recognize or respond to patient deterioration [6]. This issue is particularly concerning in non-ICU environments, where higher nurse-to-patient ratios require nurses to divide their attention among multiple patients, making timely identification of patient deterioration more challenging [7]. As van Galen et al [8] reported, 46% of 49 unplanned ICU transfers were linked to insufficient monitoring by nurses, highlighting the urgent need for enhanced monitoring strategies to support timely identification of at-risk patients in general wards.

The early warning score (EWS) is a widely used tool for early detection of patient deterioration in non-ICU settings [9]. It assigns weighted scores to vital signs (eg, respiratory rate, oxygen saturation, blood pressure, heart rate, temperature, and consciousness) to assess severity and guide interventions [9]. Studies have shown that the EWS can effectively identify patient deterioration, potentially reducing mortality rates in general wards [10,11]. The systematic review by Smith et al [11] of 21 retrospective studies in academic hospitals, primarily involving patients from medical and surgical wards, found that applying EWS was associated with a reduction in hospital mortality rates from nearly 50% to 40% and reduced cardiac arrest rates from 50% to 35%. However, studies also highlight that the effectiveness of the EWS can be compromised by inaccuracies in recorded data and its inability to capture immediate, real-time changes in vital signs [12,13]. In many general wards, nurses measure vital signs infrequently due to high patient-to-nurse ratios (especially compared with ICU settings) and intense workloads, which leave significant gaps during which critical signs of patient deterioration may be missed [12,13]. Incomplete and delayed data are associated with 19% of EWS inaccuracies in the United Kingdom and untimely responses in 75% of high-risk cases [14].

To help nurses improve the accuracy and timeliness of patient monitoring, real-time continuous monitoring systems with deterioration alerts have been proposed. Using wearable sensors or bedside monitors, these systems provide continuous data on key physiological parameters and generate alerts when out-of-range values are detected [15,16]. Multiple reviews have examined the effectiveness of these systems in non-ICU settings. Two reviews highlight positive outcomes: Downey et al [15] reviewed 24 studies involving over 40,000 patients and reported benefits such as reduced ICU transfers, shorter hospital stays, and significant cost savings. Similarly, Sun et al [16], in their meta-analysis of 14 studies, found that patients under continuous monitoring had a 39% lower risk of mortality compared to those with intermittent monitoring. However, other reviews have not found that continuous monitoring improves patient outcomes. Cardona-Morell et al [17] conducted a systematic review and meta-analysis of 22 clinical studies, involving sample sizes ranging from 16 to 64,661, and found no significant differences in the proportion of high-risk patients requiring urgent attention, preventing serious adverse events, cardiac arrests, or reducing ICU transfers. Similarly, the meta-analysis by Areia et al [18] reported potential reductions in ICU admissions and complications but found no differences compared to standard monitoring.

The differences in results across these reviews may be attributed to several factors. First, the design and functionality of continuous monitoring devices and their deterioration alerting systems vary, leading to differences in how alerts are generated and how clinicians interact with the devices. For example, Areia et al [18] focused solely on wearable devices, while Cardona-Morrell et al [17] included both wearable devices and bedside monitors. Second, the sample size and composition differed across studies. Downey et al [15] included 24 studies covering 40,274 patients and 59 ward staff across 9 countries, whereas Sun et al [16] analyzed 14 studies involving only 14,880 patients without including staff perspectives, resulting in a smaller and more limited participant base. Finally, these reviews primarily focused on device effectiveness, with limited attention to factors such as patient satisfaction and clinicians’ adoption preferences, while all emphasized the need for future research on usability factors to enhance the devices’ adoption and overall effectiveness [15-18]. Collectively, these findings highlight the limited focus on usability in current reviews, underscoring the need for further investigation into usability barriers and their impact on device usage.

Usability is a key factor in the adoption and successful use of health care technology, as it directly influences whether systems can achieve their intended goals, such as improving patient outcomes. Notably, these devices do not function in isolation. While they cannot make decisions, they provide essential information that shapes clinical judgment. Therefore, careful consideration of how these devices are integrated into clinical practice is critical. In this sense, the device’s usability reflects both its inherent attributes (eg, battery life, sensor design, and interface clarity) and its application within the broader health care context, where clinicians interpret data, make treatment decisions, and adapt workflows accordingly. Poor usability, such as unclear interfaces, false alarms, or complex workflows, can lead to clinician disengagement, reducing trust and limiting the system’s impact on clinical decisions and care processes [19].

According to the International Organization for Standardization (ISO), usability is defined as “the extent to which a product can be used by specified users to achieve specified goals with effectiveness, efficiency, and satisfaction in a specified context of use” [20]. ISO Standard 9241-11:2018 further breaks usability into 3 dimensions: effectiveness, which refers to the accuracy and completeness with which users achieve their goals; efficiency, the resources required to achieve these goals; and satisfaction, the comfort and acceptability of the system to its users [20]. As highlighted in the previous paragraph, studies on continuous monitoring and alerting systems in non-ICU settings often emphasize effectiveness while neglecting other critical factors, such as satisfaction and efficiency [15-18]. Therefore, this review aimed to map existing evidence on the usability of continuous monitoring devices with deterioration alerting systems.

## Methods

The review followed the Joanna Briggs Institute methodology for scoping reviews. A scoping review was chosen because it enables mapping of heterogeneous evidence across varied definitions of “usability,” ensuring that all aspects of the concept are covered. The review also adhered to the PRISMA-ScR (Preferred Reporting Items for Systematic Reviews and Meta-Analyses extension for Scoping Reviews) guidelines for transparent reporting.

The objectives of the review were framed using a Population, Concept, and Context framework as follows:

- Participants: the review includes studies involving clinicians working in non-ICU adult care settings, focusing on those who interact directly with continuous monitoring devices with deterioration alerting capabilities.
- Concept: the concept evaluated is the usability (effectiveness, efficiency, and satisfaction) of continuous monitoring devices that monitor vital signs and trigger alerts. These devices must demonstrate how they improve clinical outcomes, enhance workflow efficiency, and increase user satisfaction.
- Context: the context includes hospital settings where these devices are applied in real-world non-ICU scenarios. Studies must describe how clinicians receive alerts and interact with these systems to ensure a comprehensive usability assessment.

No published protocol or registration was in place before this study.

### Search

The search was conducted in published electronic databases up to November 13, 2024, including MEDLINE, Embase, Evidence-Based Medicine Reviews, Web of Science, and IEEE Xplore databases. Gray literature was searched through Web of Science.

The search strategy, incorporating the Population, Concept, and Context framework and MeSH (Medical Subject Headings) terms, was refined using search keywords from previous reviews on continuous monitoring devices with deterioration alerting systems’ capabilities [15-18,21].

A sample search strategy for MEDLINE was as follows:

(“adult care unit*” OR “general ward*” OR hospitalization OR inpatient) AND (continuous* OR “real-time” OR remote* OR wearable* OR sensor* OR monitor*) AND (“early warning score*” OR “track and trigger” OR “deterioration alert*” OR “deterioration alarm*” OR “deterioration warn*”)

The final search terms and database results are provided in Multimedia Appendix 1.

### Eligibility Criteria

The review included studies that evaluated the usability (of continuous monitoring devices with deterioration alerting, operated by clinicians in non-ICU hospital settings. Usability, as defined by ISO 9241-11:2018, consists of 3 key dimensions:

- Effectiveness: the accuracy and completeness with which users achieve their goals (eg, impact on ICU transfers, hospital stays, and patient outcomes).
- Efficiency: the resources required to achieve these goals (eg, alarm burden, false alert rates, and nursing workload).
- Satisfaction: the comfort and acceptability of the system to its users (eg, clinician and patient perceptions of comfort and acceptability).

The review focused on wearable sensors and bedside monitors that automatically track vital signs—including heart rate, blood pressure, respiratory rate, oxygen saturation, and temperature—at intervals of no more than 15 minutes. Studies were included if they described how clinicians received alerts, as this helps assess the impact of alerts and devices on clinical practice. Exclusion criteria omitted studies conducted in ICU settings; those in which clinicians—especially nurses, who are primarily responsible for patient monitoring—were blinded to alerts or not included in the research; studies involving participants under the age of 18 years; non-English publications; and review or protocol papers. The full criteria are provided in Textbox 1.

To ensure consistency in applying the inclusion and exclusion criteria during the study selection process, a dual-reviewer approach was used. The second reviewer independently evaluated 33% of studies at each review stage, including title/abstract screening and full-text review. If discrepancies exceeded 25%, the criteria were revised, and the screening process was repeated until the discrepancy rate fell below this threshold. Conflicts of less than 25% were resolved through consensus or consultation with a third reviewer. Additionally, reference lists of included studies were examined to identify further relevant studies meeting the inclusion criteria.

Textbox 1. **Inclusion and exclusion criteria.**

**Inclusion criteria:**

- Studies evaluating the usability (effectiveness, efficiency, and satisfaction) of continuous monitoring devices with deterioration alerting in hospital settings.
- Studies demonstrating the use of continuous monitoring devices with deterioration alerting in patient monitoring: alerts must be triggered by monitoring one or more vital signs such as heart rate, blood pressure, respiratory rate, oxygen saturation, or temperature.
- Studies that describe how clinicians receive alerts.

**Exclusion criteria:**

- Studies not published in English.
- Studies that did not apply continuous monitoring devices with deterioration alerting in non–intensive care unit (ICU) hospital settings or failed to clearly segregate data from non-ICU and ICU settings.
- Studies in which clinicians do not receive or acknowledge alerts: exclude studies where clinicians are kept unaware of alerts from monitoring devices (ie, clinicians were “blinded” to alerts, or the device did not generate alerts, unless clinicians had another way to receive the alerts, such as from ringing notifications or a central station).
- Studies stating that they involved participants younger than 18 years.
- Review or protocol papers: systematic reviews, meta-analyses, narrative reviews, and research protocols are excluded to focus solely on original research and first-hand data regarding device usability.

### Data Extraction

A structured data extraction form (Multimedia Appendix 2) was used to collect details on citations (author and year), study characteristics (participants, setting, design, methods, and country), device features (type, function, alerts, and application), and usability aspects (effectiveness, efficiency, satisfaction, and barriers).

First, a pilot data extraction phase involved both reviewers independently reviewing 2 included studies. Second, after independent extraction, both reviewers convened to discuss their findings. Discrepancies were resolved through discussion, and any unresolved issues were referred to a third reviewer. Third, following alignment on the pilot studies, the first reviewer extracted data from the remaining studies. The second reviewer then reviewed these extractions to verify consistency with the initial agreement and the standard definitions discussed. Remaining conflicts were resolved through consensus or consultation with a third reviewer.

### Synthesis

Studies were summarized and categorized based on key attributes, including publication year, country, study design, and research aim. First, these categorizations mapped the current research landscape and identified the distribution of studies across various contexts. For example, the percentage of studies conducted in different wards was reported to highlight where these devices are most frequently used. Second, the extracted data were categorized by common characteristics, such as device type and alert type. Finally, a narrative synthesis was conducted, thematically categorizing findings to align with the research question and objectives. Studies were grouped by their focus on usability (effectiveness, efficiency, and satisfaction) and barriers to usability, summarizing their findings, such as the accuracy of devices in detecting patient deterioration, categorized under effectiveness.

## Results

### Search Outcome

**Figure 1.**
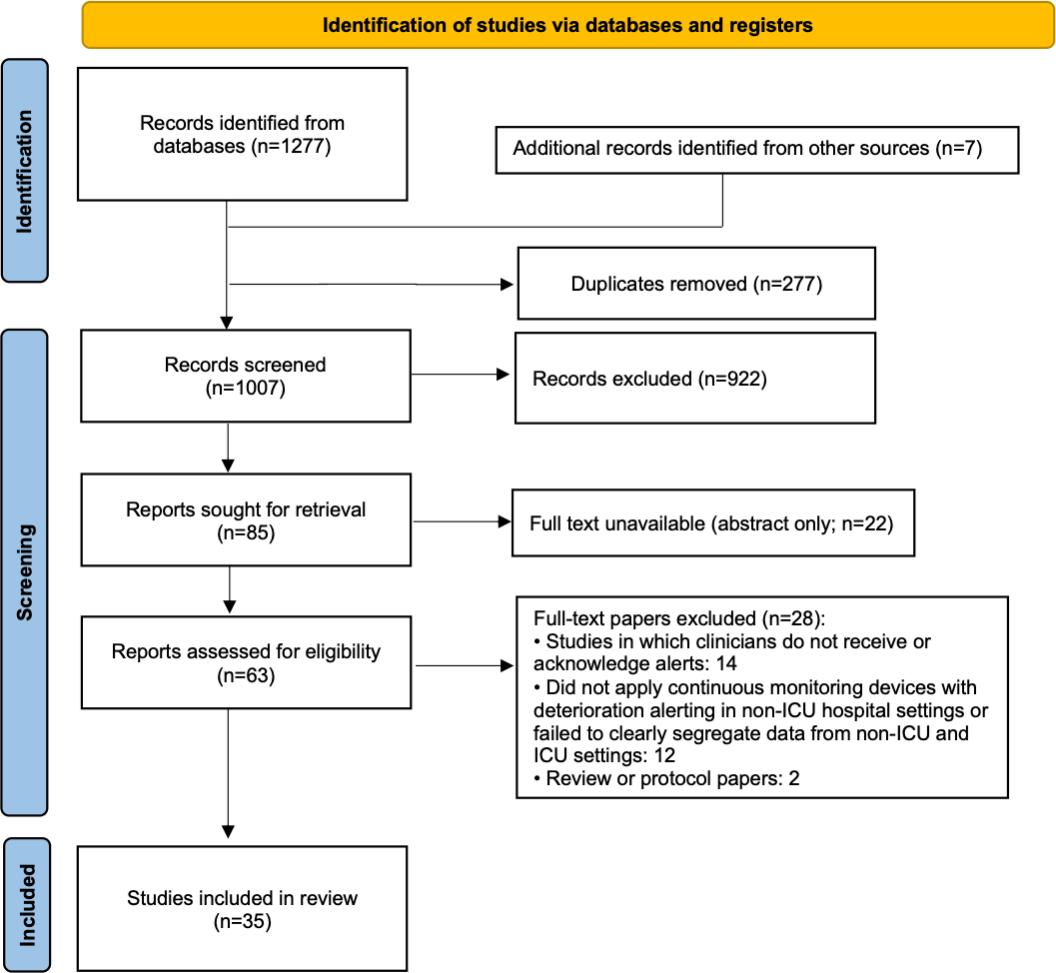
PRISMA (Preferred Reporting Items for Systematic Reviews and Meta-Analyses) flowchart.

The search identified 1284 paper citations. After excluding duplicate records, 1007 records were deemed eligible for screening. A total of 63 studies were selected based on abstracts and underwent full-text review. After applying the inclusion and exclusion criteria, 35 studies were selected for this review. Figure 1 provides the study selection process in accordance with the PRISMA (Preferred Reporting Items for Systematic Reviews and Meta-Analyses) guidelines (the PRISMA checklist is provided in Checklist 1).

### Overview of Included Studies

An overview of the included studies, including study design, study devices, interventions, comparison groups, and outcomes measured, is summarized in Table 1. Overall, the 35 studies reported outcomes for a total of 65,029 patients and 323 clinicians in 8 countries. Multimedia Appendix 3 provides a summary of the included studies.

**Table 1.** Summary of the included studies.

Author	Study aim type	Study design	Sample size	Alert mechanism type	Device type
Becking-Verhaar et al [22]	Implementation and feasibility of continuous monitoring systems	Cross-sectional survey	58 nurses	Threshold alert	Wearable devices
Bellomo et al [23]	Impact on clinical outcomes and patient safety	Observational study	18,305 patients (9617 before intervention and 8688 after intervention)	EWS^a^ base alerts	Bedside monitors
Blankush et al [24]	Technological evaluation and alarm strategies	Observational study	133 patients	EWS base alerts	Bedside monitors
Brown et al [25]	Impact on clinical outcomes and patient safety	Randomized controlled trial	Baseline cohort: 1535 (control) and 1433 (intervention); postimplementation cohort: 2361 (control) and 2314 (intervention)	Threshold alerts	Bedside monitors
Downey et al [26]	Nurses’ and patients’ perspectives and experiences	Randomized controlled trial	226 patients randomized (140 to continuous monitoring and 86 to intermittent monitoring)	Threshold alerts	Wearable devices
Downey et al [27]	Comparison with episodic monitoring	Observational study	12 patients	Threshold alerts	Wearable devices
Downey et al [28]	Implementation and feasibility of continuous monitoring systems	Randomized controlled trial	136 patients	Threshold alerts	Wearable devices
Eddahchouri et al [29]	Comparison with episodic monitoring	Observational study	Baseline cohort: 2466 admissions and intervention cohort: 2303 admissions	Threshold alerts	Wearable devices
Gazarian [30]	Nurses’ and patients’ perspectives and experiences	Prospective, descriptive, observational study	57 patients observed, 37 on continuous ECG^b^ monitoring; 9 nurses	Threshold alerts	Wearable devices
Hravnak et al [31]	Technological evaluation and alarm strategies	Observational study	629 patients (323 in phase I and 306 in phase III)	AI-based^c^ alerts	Bedside monitors
Hravnak et al [32]	Technological evaluation and alarm strategies	Observational study	326 patients	AI-based alerts	Bedside monitors
Joshi et al [33]	Technological evaluation and alarm strategies	Observational study	50 patients	Threshold alerts	Wearable devices
Klumpner et al [34]	Technological evaluation and alarm strategies	Observational study	64 monitored rooms	EWS base alerts	Bedside monitor + wearable devices
Kuznetsova et al [35]	Implementation and feasibility of continuous monitoring systems	Observational study	35 (preimplementation: 13 and postimplementation: 22) clinicians	Threshold alerts	Bedside monitors
Leenen et al [36]	Implementation and feasibility of continuous monitoring systems	Observational study	12 nurses	Threshold alerts	Wearable devices
Leenen et al [37]	Implementation and feasibility of continuous monitoring systems	Observational study	30 patients and 23 nurses	Threshold alerts	Wearable devices
McGrath et al [38]	Impact on clinical outcomes and patient safety	Observational study	2 surgical units and 71 total beds	Threshold alerts	Wearable devices
McGrath et al [39]	Impact on clinical outcomes and patient safety	Observational study	Preimplementation: 4324 patient days and postimplementation: 4382 patient days	Threshold alerts	Wearable devices
McGrath et al [40]	Technological evaluation and alarm strategies	Observational study	General care units, including a 36-bed orthopedics unit and other surgical and medicine units; the exact number of participants was not specified, but the system covered more than 200 inpatient beds.	Threshold alerts	Wearable devices
Mestrom et al [41]	Impact on clinical outcomes and patient safety	Observational study	Control group: 320 patients and intervention group: 274 patients	EWS base alerts	Bedside monitor
Paul et al [42]	Impact on clinical outcomes and patient safety	Randomized controlled trial	Control group: 126 patients and intervention group: 124 patients	Threshold alerts	Wearable devices
Peelen et al [43]	Nurses’ and patients’ perspectives and experiences	Observational study	1529 patients	AI-based alerts	Wearable devices
Pollack et al [44]	Implementation and feasibility of continuous monitoring systems	Observational study	298 patients	Threshold alerts	Wearable devices
Posthuma et al [45]	Implementation and feasibility of continuous monitoring systems	Observational study	742 patients (515 intermittent monitoring and 227 continuous monitoring)	Threshold alerts	Wearable devices
Sigvardt et al [46]	Nurses’ and patients’ perspectives and experiences	Observational study	20 patients	Threshold alerts	Wearable devices
Stellpflug et al [47]	Impact on clinical outcomes and patient safety	Observational before-and-after study	547 patients during the intervention period and 27 nurses	Threshold alerts	Wearable devices
Subbe et al [48]	Impact on clinical outcomes and patient safety	Observational before-and-after study	Control: 2139 patients and intervention: 2263 patients	Threshold alert	Bedside monitors + wearable devices
Taenzer et al [49]	Impact on clinical outcomes and patient safety	Observational before-and-after study	Preimplementation: 3118 discharges (intervention unit), 1260 (comparison unit 1), 2628 (comparison unit 2); postimplementation: 2841 discharges (intervention unit), 1162 (comparison unit 1), 2389 (comparison unit 2); 60 nurses	Threshold alert	Wearable devices
Un et al [50]	Implementation and feasibility of continuous monitoring systems	Observational study	34 patients	AI-based alerts	Wearable devices
van Goor et al [51]	Implementation and feasibility of continuous monitoring systems	Observational before-and-after study	209 patients (93 intermittent monitoring and 121 continuous monitoring)	Threshold alert	Wearable devices
van Rossum et al [52]	Comparison with episodic monitoring	Observational retrospective study	39 patients	Threshold alerts	Wearable devices
Verrillo et al [53]	Comparison with episodic monitoring	Observational study	Preintervention: 427 patients and intervention: 422 patients	Threshold alerts	Wearable devices
Watkins et al [54]	Impact on clinical outcomes and patient safety	Prospective observational study	236 patients and 24 nurses	AI-based alerts	Wearable devices
Weenk et al [55]	Comparison with episodic monitoring	Randomized controlled trial	60 patients	EWS-based alerts	Wearable devices
Weller et al [56]	Impact on clinical outcomes and patient safety	Prospective, observational study	736 patients, 23 nurses, and 20 nursing assistants	Threshold alert	Wearable devices

a EWS: early warning score.

b ECG: electrocardiogram.

c AI: artificial intelligence.

### Characteristics of Included Studies

The overview of the studies’ characteristics is summarized in Table 2. The aim of the included studies was categorized into 5 main subjects: implementation and feasibility of continuous monitoring systems (n=9, 24%) [22,28,35-37,44,45,50,51], comparison with episodic monitoring (n=5, 14%) [27,29,52,53,55], impact on clinical outcomes and patient safety (n=11, 31%) [23,25,38,39,41,42,47-49,54,56], nurses’ and patients’ perspectives and experiences (n=4, 11%) [26,30,43,46], and technological evaluation and alarm strategies (n=6, 17%) [24,31-34,40]. The included studies were predominantly from 3 countries: the United States (n=16, 46%) [24,25,30-32,34,35,38-40,44,47,49,53,54,56], the Netherlands (n=10, 29%) [22,29,36,37,41,43,45,51,52,55], and the United Kingdom (n=5, 14%) [26-28,33,48]. The most common study design was observational (n=28, 80%) [23,24,27,29-41,43,44,46-54,56]. Additionally, 20 of the included studies (57%) were categorized as nonrandomized trials [23-25,29,31-33,38,39,41,43,44,47-52,54,56]. Mixed-method studies were the least frequently used, with 2 studies (6%) [22,40]. Seventy-four percent of the included studies had comparison groups (n=26), with before-and-after implementation comparisons (n=11, 31%) [23-25,31,32,35,39,40,49,51,56] and comparisons with intermittent monitoring (n=12, 34%) [26,28,29,34,38,41-43,45,46,48,53] being most commonly used. Sixty-three percent of studies used methods combining electronic health records (EHRs), observations, clinical trials, and experiments (n=22) [23-25,29-34,39,41-43,45,46,48-53,56]. The studies most commonly involved surgical-related wards (n=18, 51%) [22,24-27,29,30,35,36,38-40,42,43,45,49,52,54] and postoperative patients (n=17, 49%) [26-30,33,37-39,41-43,45,49,52-54].

**Table 2.** Overview of characteristics of included studies.

Categories	Studies, n (%)	References
Study aim (n=35)		
Implementation and feasibility of continuous	9 (26)	[22,28,35-37,44,45,50,51]
Comparison with episodic monitoring	5 (14)	[27,29,52,53,55]
Impact on clinical outcomes and patient safety	11 (31)	[23,25,38,39,41,42,47-49,54,56]
Nurses’ and patients’ perspectives and experiences	4 (11)	[26,30,43,46]
Technological evaluation and alarm strategies	6 (17)	[24,31-34,40]
Country (n=35)		
United States	16 (46)	[24,25,30-32,34,35,38-40,44,47,49,53,54,56]
Netherlands	9 (29)	[22,29,36,37,41,43,45,51,52,55]
United Kingdom	5 (14)	[26-28,33,48]
United States, Europe, and Australia	1 (3)	[23]
Others	3 (9)	[42,46,50]
Study design (n=35)		
Observational study (prospective and retrospective before-and-after)	28 (71)	[23,24,27,29-41,43,44,46-54,56]
Randomized control trial	6 (12)	[25,26,28,42,45,55]
Cross-sectional survey	1 (3)	[22]
Comparison group (n=35)		
Before-and-after implementation comparison	11 (31)	[23-25,31,32,35,39,40,49,51,56]
Comparisons with intermittent monitoring	12 (34)	[26,28,29,34,38,41-43,45,46,48,53]
Comparisons with baseline data	1 (3)	[47]
Comparison between the monitor and alert method	2 (6)	[52,55]
No comparison	9 (26)	[22,27,30,33,36,37,44,50,54]
Clinical settings (n=35)		
Surgical-related wards	18 (51)	[22,24-27,29,30,35,36,38-40,42,43,45,49,52,54]
General wards	7 (20)	[23,37,46-48,51,53]
Surgical and internal unit	1 (3)	[55]
Others	9 (26)	[28,31-34,41,44,50,56]
Patient type (n=35)		
Postoperative	17 (49)	[26-30,33,37-39,41-43,45,49,52-54]
General medical, trauma, and surgical patients	1 (3)	[25]
Respiratory includes (COVID-19)	4 (11)	[24,48,50,51]
Other	6 (17)	[31,32,34,46,47,56]
Not specified	7 (20)	[22,23,35,36,40,44,55]
Device type (n=35)		
Wearable devices	26 (74)	[22,26-30,33,36-40,42-47,49-56]
Bedside monitors	5 (14)	[23-25,35,41]
Bedside monitors + wearable devices	4 (11)	[31,32,34,48]
Alert path (n=35)		
Alerts at central stations or system	14 (40)	[22,23,29-32,35,38,41,43,44,47,50,54]
Alert to the central station or system and clinicians’ phones or mobile devices	21 (60)	[24-28,33,34,36,37,39,40,42,45,46,48,49,51-53,55,56]
Alert mechanism type (n=35)		
Threshold alerts	25 (71)	[22,25-30,33,35-40,42,44-49,51-53,56]
Early warning score–based alerts	5 (14)	[23,24,34,41,55]
Artificial intelligence–based alerts	5 (14)	[31,32,43,50,54]

### Characteristics of Clinicians

Ten (29%) of the included studies reported clinicians’ characteristics involved in using continuous monitoring devices with deterioration alerting [22,30,35-37,47,49,53,54,56] (Multimedia Appendix 4). Seven studies reported incomplete characteristics [22,36,47,49,53,54,56], and 4 studies provided sample size and profession [47,49,54,56]. The available data indicated more female than male clinicians, with mean ages between 27 and 30 years. Most clinicians had 5-10 years of work experience, with nurses being the majority of users (Multimedia Appendix 5).

### Overview of Study Devices

The devices used can be classified into wearable devices and bedside monitors. Wearable devices, worn directly by the patient, monitor vital signs continuously while allowing patient mobility. The most commonly used wearable devices were the SensiumVital patch (Sensium Healthcare; n=8, 23%) [26-28,33,36,37,45,52] and ViSi Mobile (Sotera Wireless Inc; n=7, 20%) [22,43,51,53-56]. The SensiumVital patch, applied to the chest, monitors heart rate, respiratory rate, and body temperature, transmitting data every 2 minutes to a central station or mobile device, with visual alerts for deviations from preset vital signs [26]. ViSi Mobile monitors heart rate, blood pressure, respiratory rate, body temperature, and oxygen saturation from the upper arm, chest, and wrist, sending visual alerts to a central monitor and the nurse’s Wi-Fi phone [56]. Bedside monitors are stationary devices placed near the patient’s bed, offering continuous monitoring within the monitor’s vicinity. The IntelliVue Guardian Solution (Philips), used in 4 studies, tracks heart rate, respiratory rate, blood pressure, body temperature, and oxygen saturation, providing visual alerts on central and bedside monitors [23,24,41,48]. It can also be used with wireless monitors on the chest, wrist, and upper arm, ensuring continuous monitoring and timely clinical actions [23,24,41,48].

The alerting mechanisms in the included studies were categorized into 3 groups: threshold alerts, EWS-based alerts, and artificial intelligence (AI)–based alerts. Threshold alerts (n=25, 71%) notify health care providers when a monitored vital sign exceeds predefined limits, typically applied to respiratory rate, oxygen saturation, heart rate, and blood pressure (with temperature less consistently included), although only a minority of studies reported explicit numerical cut-off values or which parameters most commonly triggered alarms [22,25-30,33,35-40,42,44-49,51-53,56]. EWS-based alerts (n=5, 14%) are generated using an aggregated score based on multiple vital signs to identify patients at risk of deterioration [23,24,34,41,55]. AI-based alerts (n=5, 14%) use algorithms to analyze vital signs and predict potential clinical deterioration [31,32,43,50,54]. In terms of delivery methods, 20 studies reported systems that sent alerts directly to clinicians’ mobile devices, including phones or pagers [24-28,33,34,36,37,39,40,42,46,48,49,51-53,55,56], while also notifying the central nurse station. The remaining studies indicated that alerts were sent exclusively to the central station, highlighting variability in alert dissemination approaches.

### Effectiveness, Efficiency, Satisfaction, and Barriers to Usability

The included studies were analyzed for their reported effectiveness, efficiency, satisfaction, and barriers to usability.

### Effectiveness

Twenty-five studies (71% of all included studies) provided relevant data on effectiveness [23,25,26,28,29,31-33,36,37,40-45,47-50,52-56]. Eight of these studies (32%) reported on rapid response team (RRT) calls: 2 studies indicated an increase [23,48], 1 study reported no significant change [53], and 5 studies observed a decrease in RRT calls [29,40,47,49,56]. ICU transfer rates were evaluated in 13 (52%) studies, with 3 studies finding no significant change [25,42,45] and 10 studies reporting a decrease [28,29,31,32,40,47-49,53,56]. Mortality rates were assessed in 12 studies, with 5 studies noting an increase in survival rates [23,31,32,48,49] and 7 studies finding no significant change [26,28,29,41,53,56]. The length of hospital stay was examined in 12 studies; 2 studies did not specify results [37,50], 5 studies found no significant change [29,41,45,49,56], and 5 studies reported a decrease [23,25,26,28,47]. Readmission rates were evaluated in 3 studies, with 1 study [41] finding no significant change and 2 studies [26,28] observing a decrease. Serious adverse events were investigated in 6 studies, with 2 studies identifying adverse events [50,52] and 4 studies reporting a decrease [31,32,48,53]. Notably, 5 studies using AI-based alerting systems demonstrated potential benefits [31,32,43,50,54], including reduced mortality and ICU transfer rates [31,32] and improved identification and reduction of SAEs [31,32,50]. However, no significant tendency was observed across other measured outcomes. Of the 4 studies that combined bedside monitors with wearable devices [31,32,34,48], 3 reported strong effectiveness in reducing mortality, ICU transfers, and SAEs [31,32,48]. However, the effectiveness of bedside monitors alone remains unclear due to the limited number of studies.

Overall, more studies suggested that RRT calls and ICU transfer rates decrease after implementing continuous monitoring devices with deterioration alerting systems. However, the effects on mortality, length of hospital stay, readmissions, and serious adverse events remain inconclusive.

### Efficiency

Twenty-six studies (74%) provided insights into efficiency issues, such as alarm frequency, false alert rate, workload impact, and time saving [22-24,30-40,42-44,46,47,49-52,54-56]. Alarm frequency was reported in 15 studies, with 4 studies indicating excessive alarms (over 4 alarms per patient per day) [24,30,36,56] and 11 studies reporting fewer than 5 alarms per patient per day [34,35,37-40,42,43,49,52,54]. The false alert rate was reported in 12 studies; 7 studies reported a high false alert rate (>40%) [24,30,36,37,43,50,52], while 5 studies reported a low false alert rate [31,34,44,49,56]. The impact on workload was investigated in 15 studies; 5 studies were unsure of the impact [22,39,47,51,56], 3 studies reported an increase in workload [24,36,54], and 7 studies observed a reduction in workload [32,34,35,38,40,46,50]. Time savings were evaluated in 8 studies, with 1 study reporting unclear results [55] and 7 studies reporting time savings [22,35,38,44,46,47,56]. Most current studies suggest that continuous monitoring devices save time, although the impact on workload is less clear. Alarm frequency generally remained below 5 alarms per patient per day, but false alarms were reported as a common and significant issue.

### Satisfaction

Satisfaction with the devices was reported in 16 (46%) studies. Comfort was reported in 8 studies, focusing only on patient perspectives. Among these, 88% (n=7) [26-28,37,42,44,47] reported that patients found the devices comfortable, while 1 study reported discomfort [22]. Acceptability was reported in 94% of the studies, with 8 studies reporting patient acceptability [22,26-28,37,39,42,49] and 9 studies reporting clinician acceptability [35,36,38,40,44,47,49,56]. High acceptability was found among both patients and clinicians, with 1 study not clearly stating clinician acceptability [53].

### Barriers to Usability

Usability barriers were formally reported in 10 studies (29% of all included studies) [22,26,27,30,35,36,39,44,47,49], while 5 studies mentioned them only in their discussion [24,28,40,42,43]. These barriers included technical issues, alarm management, patient comfort and experience, training and knowledge needs, and workflow integration challenges (Table 3).

**Table 3.** Summary of barriers to usability (n=10, 29% of all studies).

Studies	Definition	Studies, n (%)
Technical issues [22,39,44]	Issues with device connectivity, battery life, sensor attachment, internet connectivity, and overall device design, along with technical difficulties and artifactual issues, such as poor lead adherence and interference, as well as the cumbersome nature of device use, including removal and reapplication for showers.	3 (20)
Alarm management [26,30,49]	Frequent and high rates of initial false alarms leading to alarm fatigue and inconvenience, inconsistent practices in managing alarms, and excessive alarms from the system, often due to malfunctioning hardware or baseline tachycardia.	3 (20)
Patient comfort and experience [26,27,36,39]	Discomfort and skin reactions from the patch, concerns about practicalities such as showering, trust issues with technology reliability, patient refusals due to discomfort, confusion, or personal reasons, and discontinued monitoring due to various factors, such as contact allergies or initiation of palliative care.	4 (27)
Training and knowledge needs [22,26,35,36,39]	The need for patient education, ongoing training and coaching for health care providers, and challenges including alarm fatigue, accuracy and trust issues, and insufficient training for secondary users are impacting the patient experience.	5 (33)
Workflow and integration issues [27,36,47,49]	Challenges with integrating the monitoring system into clinical workflows, managing alarm burdens, interpreting vital sign trends, ensuring seamless integration with hospital systems, and the potential reduction in face-to-face nursing contact.	4 (27)

Technical issues were identified in 3 studies and included problems with device connectivity, battery life, sensor attachment, internet connectivity, and overall device design, along with artefactual issues such as poor lead adherence (ie, leads detaching frequently and affecting continuous monitoring), interference, and the cumbersome nature of device use, including removal and reapplication for showers [22,39,44]. Alarm management was a concern in 3 studies [26,30,49], with frequent and high rates of initial false alarms leading to alarm fatigue and inconvenience, inconsistent practices in managing alarms, and excessive alarms often resulting from malfunctioning hardware or baseline tachycardia. Unlike efficiency measures, which assess the total number of alarms and false alarm rates quantitatively, these studies highlighted usability-related challenges, such as how clinicians respond to alarms, their perceived reliability of alerts, and whether excessive alarms led to desensitization or delayed responses to actual critical alerts. Additionally, alarm issues were often linked to hardware malfunctions or patient baseline conditions (eg, persistent tachycardia triggering unnecessary alerts), which further complicated clinical workflows and increased clinician frustration. Patient comfort and experience were highlighted in 4 studies [26,27,36,39], discussing discomfort and skin reactions from the patch, practical concerns such as showering, trust issues with technology reliability, patient refusals due to discomfort, confusion, or personal reasons, and discontinued monitoring due to contact allergies or the initiation of palliative care. Training and knowledge needs were emphasized in 5 studies [22,26,35,36,39], underscoring the need for patient education, ongoing training, and coaching for health care providers, with challenges including alarm fatigue, accuracy and trust issues, and insufficient training for secondary users impacting the patient experience. Workflow and integration issues were reported in 4 studies [27,36,47,49], noting challenges with integrating the monitoring system into clinical workflows, managing alarm burdens, interpreting vital sign trends, ensuring seamless integration with hospital systems, and the potential reduction in face-to-face nursing contact. Finally, only 6 studies systematically surveyed usability barriers from clinicians’ perspectives, collecting data through interviews, surveys, or by directly including clinicians as participants [22,30,35,36,47,49].

## Discussion

### Principal Findings

This scoping review aimed to map the evidence on the usability of continuous monitoring devices with deterioration alerting in non-ICU settings, according to the ISO standard. It focused on effectiveness, efficiency, satisfaction, and the barriers affecting usability. Current review findings suggest that while most research supports the effectiveness and efficiency of these devices, evidence regarding satisfaction and barriers to usability remains limited, with usability barriers receiving the least attention. Through the limited evidence, 5 key barriers to usability were identified in this review: (1) technical issues (eg, connectivity and battery limitations), (2) alarm management challenges (eg, false alarms and alarm fatigue), (3) patient comfort concerns (eg, skin irritation), (4) training gaps for clinicians, and (5) workflow integration difficulties. Considering the impact of barriers to usability on effectiveness, efficiency, and satisfaction, a critical gap in the literature is highlighted [19]. Future research should prioritize investigating usability barriers by examining patient and clinician experiences and developing interventions to overcome implementation challenges.

The findings of this review suggest that continuous monitoring devices with deterioration alerting are associated with reductions in RRT calls and ICU transfer rates in some studies, supporting previous reviews. Cardona-Morrell et al [17] and Sun et al [16] also reported significant reductions in cardiac arrest calls and rescue events associated with continuous monitoring. However, mortality outcomes remain inconclusive, likely due to variability in study designs, small sample sizes, and differences in patient populations or clinical settings. Similarly, Cardona-Morrell et al [17] and Areia et al [18] found no significant impact on mortality, while Sun et al [16] reported a 39% reduction in mortality risk, making it the only review with a significant finding. Interestingly, previous reviews all reported nonsignificant reductions in ICU transfers, which contrasts with the findings of this review [15-18]. These findings collectively suggest that continuous monitoring devices may improve patient outcomes; however, further research is needed to provide more evidence supporting their comprehensive benefits.

The efficiency of continuous monitoring devices with deterioration alerting is supported by evidence indicating time savings and manageable alarm frequencies. Most studies report fewer than 5 alarms per patient per day, which is generally acceptable to clinicians [56]. However, false alarms remain a significant concern, with some studies reporting rates exceeding 40% following device implementation. This issue has been highlighted in previous systematic reviews, which emphasize the need for improved alert accuracy, as false alarms are consistently identified as a usability barrier requiring further research and refinement [15,16,18]. Additionally, the impact of these devices on workload remains inconclusive, as findings vary across studies—some report a reduction in workload, others an increase, while some remain uncertain. Downey et al [15] noted that nurses who received proper training and felt confident using the technology experienced less workload strain, whereas a lack of familiarity led to disengagement and a perceived increase in workload. Given these inconsistencies, further research is needed to establish clearer trends regarding the workload impact of continuous monitoring devices.

Satisfaction with continuous monitoring devices is notably high, with both patients and clinicians reporting positive experiences. Most studies indicate that patients perceive these devices as comfortable and acceptable. While previous reviews have focused less on satisfaction metrics, Downey et al [15] provide valuable insights, noting that patients and clinicians recognize the clinical benefits of these devices and express willingness to adopt them due to enhanced patient safety. These findings align with the current review’s conclusions regarding high satisfaction levels.

The barriers to usability associated with continuous monitoring devices with deterioration alerting systems in non-ICU settings are multifaceted, which prior reviews rarely address. Technical issues found in this review, such as unreliable Wi-Fi connectivity and sensors detaching from patients (triggering nonactionable alarms), align with findings from Leenen et al [21], whose review of 13 wearable devices highlighted design limitations—including partial wiring in supposedly “wearable” systems that restrict patient mobility and generate clinically irrelevant alerts. In addition, patient discomfort and experience, manifested as skin irritation, mobility restrictions, and distrust in device reliability, have been widely recognized as a barrier in existing literature [15-18]. Furthermore, alarm management challenges, particularly false alarms, exacerbate clinician workload and desensitization, a concern echoed across previous review studies [15-18].

Notably, this review identifies training gaps and workflow integration challenges as critical yet underreported usability barriers that were not emphasized in earlier literature [15-18]. Inadequate education for both patients and clinicians—particularly regarding alarm interpretation, device operation, and troubleshooting—amplifies usability challenges and undermines system effectiveness. These findings align with Chaniaud et al [57], who suggest that even basic, widely adopted home devices (eg, blood pressure monitors and pulse oximeters) require robust user education to achieve their full potential. This highlights the universal importance of training programs tailored to device complexity and user expertise. Similarly, poorly integrated systems disrupt clinical workflows. For example, devices that lack seamless connectivity with existing EHR systems force clinicians to manually reconcile data, increasing staff burden and reducing time for direct patient care [49]. Collectively, these barriers highlight the necessity of holistic solutions that address not only technical performance but also human-centered design (eg, prioritizing patient comfort and clinician workflow efficiency) and system interoperability to maximize the potential of continuous monitoring.

One key aspect of the adoption and use of continuous monitoring systems is the critical role of clinicians who are responsible for implementing and managing these technologies. According to these findings, nurses make up the majority of front-line users, managing continuous monitoring, interpreting alerts, and overseeing these technologies in non-ICU environments. This highlights the need to prioritize nurses’ needs in the design and integration of continuous monitoring with deterioration alert systems. However, a significant limitation in the literature is the disproportionate focus on patient experiences compared to clinician-related factors. This imbalance is problematic because clinicians’ acceptance and effective use of technology are pivotal to integrating these systems successfully into clinical workflows. Few studies have examined usability barriers from the clinicians’ perspective or provided detailed data on factors such as familiarity with devices, willingness to adopt new technologies, and demographic characteristics (eg, age, gender, and experience). In fact, clinicians’ attributes can significantly influence usability, user behavior, and overall adoption of these systems [58]. Or et al [58] demonstrate that clinicians who trust and are willing to use technology are more likely to adopt EHR systems in their practice. These findings align with Carayon and Hoonakker [59], who argue that clinicians—as both implementers and end users—are as critical as system designers in determining health IT effectiveness. Their review underscores that neglecting clinician-specific barriers risks poor adoption and suboptimal outcomes. Addressing these gaps is essential for ensuring the long-term viability of continuous monitoring technologies and highlights the urgent need for studies that prioritize clinician-centered usability metrics.

Finally, this review adds several key points compared to previous reviews. First, AI-based alerting systems remain underused and are not yet well tested in these studies, indicating a need for further research to establish their effectiveness for future applications. A systematic review by Muralitharan et al [60] suggests that AI-based alerting methods perform better than threshold alerts and EWS-based alerts; however, this scoping review found limited practical application of AI-based alerting, underlining the necessity for additional clinical trials. Second, most studies on continuous monitoring devices with deterioration alerting are concentrated in the United States and are primarily conducted in surgical units with postoperative patients. This suggests that the application of these devices is currently limited to specific clinical settings, patient populations, and geographic regions. However, continuous monitoring systems have been highlighted as promising remote patient monitoring solutions that can save clinicians time by alleviating staffing burdens and improving patient safety—particularly in light of staffing shortages in both limited-, low-, and middle-income countries [61]. Third, implications for smaller community hospitals and lower-resource wards also warrant consideration. Most of the implementations identified in this review were in larger, well-resourced centers, whereas prior work has highlighted that higher nurse-to-patient ratios in general wards and human-related monitoring failures are important contributors to delayed recognition of deterioration [7,8]. In settings where 1 nurse cares for more patients and technical support is limited, the additional alarms and infrastructure required for continuous monitoring may therefore have different consequences for workload, alarm fatigue, and value for money; although earlier reviews have described potential cost savings in selected high-resource wards, economic outcomes were rarely reported in this review’s included studies, underscoring the need for formal economic evaluations—particularly in community and resource-constrained hospitals—before large-scale implementation [15,61]. These gaps highlight the importance of expanding research to assess the generalizability of continuous monitoring technologies by exploring diverse clinical environments and rigorously evaluating emerging AI-based alerting systems.

### Limitations

This review is limited to studies published in English, potentially excluding relevant non-English publications and unpublished studies. The focus on usability (effectiveness, efficiency, and satisfaction) excludes studies that did not mention or examine these aspects. Only studies providing primary data were included, potentially omitting those reporting secondary data, such as systematic reviews and meta-analyses, or unpublished studies. Due to significant variations in methodology, objectives, and reported data among the included studies, a meta-analysis was not feasible. The search strategy excluded studies conducted in the ICU or nonhospital services, focusing deliberately on noncritical adult care units to investigate usability in these settings. This may have omitted relevant studies that include both ICU and non-ICU settings. Furthermore, as a scoping review, this study aimed to include a broad range of studies without excluding any based on quality. Finally, usability was measured with heterogeneous, study-specific items, and no study used a validated instrument (eg, System Usability Scale), precluding cross-system benchmarking or pooled usability scores. Alert parameters and thresholds were inconsistently reported, limiting the comparability of alarm burden across devices and protocols.

### Conclusion

Continuous monitoring devices with deterioration alerting systems are increasingly recognized as valuable tools for preventing patient deterioration in non-ICU settings. However, their successful implementation hinges on a comprehensive understanding of usability (encompassing effectiveness, efficiency, and satisfaction) and the barriers influencing real-world adoption. This review indicates that these devices can reduce RRT calls and ICU transfers, save time, and maintain manageable alarm frequencies while achieving high user satisfaction. However, significant usability barriers remain, including technical issues, alarm management challenges, patient discomfort, and insufficient training and workflow integration. Moreover, most existing studies focus on effectiveness and efficiency, leaving satisfaction and broader usability factors understudied. Additionally, research has predominantly focused on patient perspectives, often neglecting clinician insights and has been limited to specific clinical contexts, patient populations, and geographic regions, which raises concerns about the generalizability of these findings. Future studies should prioritize usability factors and expand to the clinicians’ usage perspective and diverse health care settings to ensure these technologies deliver equitable, scalable improvements in patient safety and optimize care delivery in non-ICU environments.

## Supplementary material

10.2196/75713 Multimedia Appendix 1 Search results up to November 2024.

10.2196/75713 Multimedia Appendix 2 Data extraction sheet.

10.2196/75713 Multimedia Appendix 3 Summary of the included studies.

10.2196/75713 Multimedia Appendix 4 Characteristics of the included studies.

10.2196/75713 Multimedia Appendix 5 Summary of the clinicians’ characteristics in the included studies.

10.2196/75713 Checklist 1 PRISMA-ScR checklist.

## Abbreviations

AI: artificial intelligence
EHR: electronic health record
EWS: early warning score
ICU: intensive care unit
ISO: International Organization for Standardization
MeSH: Medical Subject Headings
PRISMA: Preferred Reporting Items for Systematic Reviews and Meta-Analyses
PRISMA-ScR: Preferred Reporting Items for Systematic Reviews and Meta-Analyses extension for Scoping Reviews
RRT: rapid response team
SAE: severe adverse event
