# Supplementary material for: The Usability of Continuous Monitoring Devices With Deterioration Alerting Systems in Noncritical Care Units: Scoping Review
Source: Interact J Med Res. 2026 Feb 10;15:e75713. doi: 10.2196/75713 (PMC12892175; doi:10.2196/75713)
Supplement: Multimedia Appendix 1 [file ijmr-v15-e75713-s001.docx]

**Attachment 1**

**Search result Until November. 2024**

| Ovid MEDLINE(R) <1946 to November Week 2 2024>  1 patient*.mp. 7856282  2 pediatric.mp. or exp Pediatrics/ 395970  3 exp Adolescent/ or adolescent*.mp. 2317326  4 infant*.mp. or exp Infant/ 1396030  5 exp Intensive Care Units/ or Intensive care unit*.mp. 188096  6 1 not 2 not 3 not 4 not 5 6402329  7 exp Monitoring, Physiologic/ or continuous monitor*.mp. 210592  8 continuous sens*.mp. 280  9 real-time sens*.mp. 473  10 real-time monitor*.mp. 8530  11 exp Wearable Electronic Devices/ or wearable sens*.mp. 23847  12 wearable monitor*.mp. 285  13 vital sign* monitor*.mp. 800  14 vital sign* sens*.mp. 48  15 7 or 8 or 9 or 10 or 11 or 12 or 13 or 14 239744  16 exp Early Warning Score/ or early warning scor*.mp. 1712  17 (track and trigger).mp. [mp=title, book title, abstract, original title, name of substance word, subject heading word, floating sub-heading word, keyword heading word, organism supplementary concept word, protocol supplementary concept word, rare disease supplementary concept word, unique identifier, synonyms, population supplementary concept word, anatomy supplementary concept word] 442  18 alert*.mp. or exp Decision Support Systems, Clinical/ 54229  19 exp Clinical Alarms/ or alarm*.mp. 32883  20 warn*.mp. 42574  21 18 or 19 or 20 125831  22 deterioration*.mp. or exp Clinical Deterioration/ 98591  23 deteriorat*.mp. [mp=title, book title, abstract, original title, name of substance word, subject heading word, floating sub-heading word, keyword heading word, organism supplementary concept word, protocol supplementary concept word, rare disease supplementary concept word, unique identifier, synonyms, population supplementary concept word, anatomy supplementary concept word] 140782  24 22 or 23 140782  25 21 and 24 2434  26 16 or 17 or 25 3842  27 6 and 15 and 26 274 |
| --- |
| Embase <1974 to 2024 November 18>  1 patient*.mp. or exp hospital patient/ 13748883  2 pediatric.mp. or exp pediatrics/ 752634  3 adolescent*.mp. or exp adolescent/ 1988418  4 infant*.mp. or exp infant/ 1417974  5 exp intensive care unit/ or intensive care unit*.mp. 401702  6 1 not 2 not 3 not 4 not 5 11928358  7 exp monitoring/ or continuous monitor*.mp. 1130490  8 continuous sens*.mp. 431  9 real-time sens*.mp. 793  10 real-time monitor*.mp. 13335  11 exp wearable sensor/ or wearable sens*.mp. 8730  12 wearable monitor*.mp. 474  13 vital sign* monitor*.mp. 1851  14 vital sign* sens*.mp. 68  15 7 or 8 or 9 or 10 or 11 or 12 or 13 or 14 1148299  16 exp early warning score/ or early warning scor*.mp. 4198  17 (track and trigger).mp. [mp=title, abstract, heading word, drug trade name, original title, device manufacturer, drug manufacturer, device trade name, keyword heading word, floating subheading word, candidate term word] 861  18 alert*.mp. 102429  19 exp alarm monitor/ or alarm*.mp. 57033  20 warn*.mp. 72060  21 18 or 19 or 20 224361  22 exp deterioration/ or deteriorat*.mp. 266272  23 21 and 22 5566  24 16 or 17 or 23 9041  25 6 and 15 and 24 768 |
| EBM Reviews - Cochrane Database of Systematic Reviews <2005 to November 13, 2024>  EBM Reviews - ACP Journal Club <1991 to October 2024>  EBM Reviews - Database of Abstracts of Reviews of Effects <1st Quarter 2016>  EBM Reviews - Cochrane Clinical Answers <October 2024>  EBM Reviews - Cochrane Central Register of Controlled Trials <October 2024>  EBM Reviews - Cochrane Methodology Register <3rd Quarter 2012>  EBM Reviews - Health Technology Assessment <4th Quarter 2016>  EBM Reviews - NHS Economic Evaluation Database <1st Quarter 2016>  1 patient*.mp. [mp=ti, ab, tx, kw, ct, ot, fx, sh, hw] 1347079  2 pediatric*.mp. [mp=ti, ab, tx, kw, ct, ot, fx, sh, hw] 44758  3 adolescent*.mp. [mp=ti, ab, tx, kw, ct, ot, fx, sh, hw] 180355  4 infant*.mp. [mp=ti, ab, tx, kw, ct, ot, fx, sh, hw] 84521  5 Intensive care unit*.mp. [mp=ti, ab, tx, kw, ct, ot, fx, sh, hw] 30403  6 1 not 2 not 3 not 4 not 5 1190793  7 continuous monitor*.mp. [mp=ti, ab, tx, kw, ct, ot, fx, sh, hw] 1107  8 continuous sens*.mp. [mp=ti, ab, tx, kw, ct, ot, fx, sh, hw] 14  9 real-time sens*.mp. [mp=ti, ab, tx, kw, ct, ot, fx, sh, hw] 19  10 real-time monitor*.mp. [mp=ti, ab, tx, kw, ct, ot, fx, sh, hw] 284  11 wearable sens*.mp. [mp=ti, ab, tx, kw, ct, ot, fx, sh, hw] 386  12 wearable monitor*.mp. [mp=ti, ab, tx, kw, ct, ot, fx, sh, hw] 45  13 vital sign* monitor*.mp. [mp=ti, ab, tx, kw, ct, ot, fx, sh, hw] 339  14 vital sign* sens*.mp. [mp=ti, ab, tx, kw, ct, ot, fx, sh, hw] 8  15 7 or 8 or 9 or 10 or 11 or 12 or 13 or 14 2153  16 early warning scor*.mp. [mp=ti, ab, tx, kw, ct, ot, fx, sh, hw] 255  17 (track and trigger).mp. [mp=ti, ab, tx, kw, ct, ot, fx, sh, hw] 94  18 alert*.mp. [mp=ti, ab, tx, kw, ct, ot, fx, sh, hw] 10232  19 alarm*.mp. [mp=ti, ab, tx, kw, ct, ot, fx, sh, hw] 2891  20 warn*.mp. [mp=ti, ab, tx, kw, ct, ot, fx, sh, hw] 3964  21 18 or 19 or 20 16473  22 deteriorat*.mp. [mp=ti, ab, tx, kw, ct, ot, fx, sh, hw] 19558  23 21 and 22 663  24 16 or 17 or 23 937  25 6 and 15 and 24 27 |
| Web of Science <2000 to Nov 19 2024>  1. ALL=(patient* ) 9,060,207  2. ALL=(pediatric*) 1,012,890  3. ALL=(adolescent*) 731,197  4. ALL=(infant*) 672,680  5. ALL=(Intensive care unit* ) 221,874  6. #1 not #2 not #3 not #4 not #5 357,687  7. ALL=(continuous monitor* ) 131,803  8. ALL=(continuous sens* ) 162,623  9. ALL=(real-time sens* ) 223,152  10. ALL=(real-time monitor* ) 162,325  11. ALL=(wearable sens* ) 55,231  12. ALL=(wearable monitor* ) 30,459  13. ALL=(vital sign* monitor* ) 19,501  14. ALL=(vital sign* sens* ) 25,925  15. #7 OR #8 OR #9 OR #10 OR #11 OR #12 OR #13 OR #14 631,724  16. ALL=(early warning scor* ) 4,825  17. ALL=(track and trigger ) 14,862  18. ALL=(alert* ) 87,489  19. ALL=(alarm* ) 101,311  20. ALL=(warn* ) 221,882  21. #18 OR #19 OR #20 397,553  22. ALL=(deteriorat* ) 322,076  23. #21 AND #22 5,024  24. #17 OR #16 OR #23 23,365  25. #6 AND #15 AND #24 85 |
| IEEE Xplore <2008 to Nov 2024>  Researchers are not permitted to use more than seven wildcards in this database. As a result, the search strategy must adapt to the words used. This search will only keep the main concept in order to narrow down and include relevant literatures. As a result, the final search phrase will be:  patient* AND (monitor* OR sens*) AND ((early warning score*) OR (( alarm* Or alert*) AND deteriorat*))  Result: 123 |
