# Supplementary material for: The Usability of Continuous Monitoring Devices With Deterioration Alerting Systems in Noncritical Care Units: Scoping Review
Source: Interact J Med Res. 2026 Feb 10;15:e75713. doi: 10.2196/75713 (PMC12892175; doi:10.2196/75713)
Supplement: Multimedia Appendix 2 [file ijmr-v15-e75713-s002.docx]

**Attachment 2 Data Extraction Sheet**

| Evidence Source Details and Characteristics | |
| --- | --- |
| Citation details | Author(s), year of publication, journal |
| Study Aim Summary  Study Aim type | Specific aim or objectives of each study |
|  | Options:   - Implementation and Feasibility of Continuous - Comparison with Episodic Monitoring - Impact on Clinical Outcomes and Patient Safety - Nurses’ and Patients’ Perspectives and Experiences - Technological Evaluation and Alarm Strategies |
| Country |  |
| Study Design | (i.e. Observational study, randomized control trial) |
| Study Design Type | Options:   - Qualitative - Randomized control trial - Non-Randomized - Quantitative Descriptive - Mixed Methods |
| Data Collection Methods | (i.e. Survey, Interview, Clinical Trials and Experiments, Observations, Electronic Health Records, Other) |
| Setting (Ward) |  |
| Participants Details |  |
| Patient Disease  Sample size (N)  Male %: Female % ratio, mean age (years)  Clinicians  Sample size (N)  Male %: Female % ratio, Age  Clinician Experience | (i.e. Mean age or Age range)  (i.e. Nurse/ Physician/ Healthcare Worker/Others)  (i.e. Mean age or Age range)  (How many years of experience? What kind of experience?) |
| Device |  |
| Device Name  Device Type  Vital Signs Measured  Applied Location  How the alert was created  Alert Type  How clinicians receive alert from Device  Does the user have training? How long is the training? | (Specific types of continuous monitoring devices studied)  (Bedside monitor/ Wearable monitor/ Bedside monitor + Wearable monitor)  HR, BP, RR, SpO2, BT  (HR: heart rate. RR: respiratory rate. SpO2: oxygen saturation BP: Blood Pressure BT: Body temperature)  (i.e. Chest, Wrist…)  (i.e. violate preset threshold and then alert (threshold alert), based on EWS then alert (EWS-based alert), The alert was based on the SpO2 and when it lower than 90% for more than 15 second the alert will create.)  Threshold alert/ EWS based alert/ AI based Alert  Which type of alert:   - Visual Alerts: Including but not limited to notifications on central monitoring stations, bedside monitors, or handheld devices such as tablets and smartphones. - Auditory Alerts: Such as alarms and ringing notifications that inform clinicians of patient deterioration. - Integrated Alerts: Alerts integrated into existing hospital systems, central station or electronic health records that trigger notifications on various devices accessible to the clinical staff.   And how this inform clinicians? (i.e. Sent message to the phone, presented on the central monitor.) |
| Details/Results extracted from source of evidence (only use the data from results section) | |
| a. Does the user have training of devices before the study?  b. How long is the training?  c. How long the device was applied in the setting? | a. Yes or No or N/A, (explain how the training proceed.)  b. Duration of the training.  c. How long has the continuous monitoring device was used by clinicians (nurses or other healthcare profession) in total in their studies. If no such information directly, the study consider the duration of the study as the time of clinicians used the continuous monitor. (i.e., in RCT how long the Intervention was used in the group. Before and after implementation study, how long the after implementation was conducted.) |
| Did the study have comparison group? | Does the study have comparison group? (No: n/a, yes: describe the comparison group?) What kind of the care they have? (i.e. Regular Care with EWS) |
| Effectiveness:  The accuracy and completeness with which specified users can achieve specified goals in the environment. Metrics include hospital mortality rates, reductions in hospital stay lengths, prevalence of serious adverse events, Rapid Response Team (RRT) calls, and ICU transfer rates. | RRT calls: i.e. decrease 20% compared to comparison group  ICU transfer rate: i.e. decrease 20% compared to comparison group  Mortality rate:  Serious adverse events:  Hospital stay length:  Readmission:  Other: Narrative Data (i.e. the monitor reduces participant transfer into ICU), or other indicators. |
| Efficiency:  The resources expended on the accuracy and completeness of goals achieved. Measures include alarm frequency per hour, rates of correct versus false alarms, nursing interventions in response to alarms, and impacts on workload and time efficiency. | Alarm Frequency (per day/ hour): i.e. 2.3 per hour (lower 20% than before)  False Alert rate:  Workload Impact:  Time Saving:  Other: Narrative Data (i.e. the monitor saves a lot of time), or other indicators. |
| Satisfaction:  The comfort and acceptability of the work system to its users and other people affected by its use. Considerations include night-time burden for patients, device comfort (e.g., devices being too heavy), and acceptability by patients and clinicians. | Comfortability:  Acceptability:  Other: Narrative Data (i.e. Nurses said they like it.), or other indicators. |
| Barrier Factors for of use | i.e. Less contact with nurses, allergic to the patch, limited the mobility…(If no specified in the results, stated it as “not specific”. |
