# Supplementary material for: The Usability of Continuous Monitoring Devices With Deterioration Alerting Systems in Noncritical Care Units: Scoping Review
Source: Interact J Med Res. 2026 Feb 10;15:e75713. doi: 10.2196/75713 (PMC12892175; doi:10.2196/75713)
Supplement: Multimedia Appendix 3 [file ijmr-v15-e75713-s003.docx]

**Table 2 (1)** **Summary of the included studies**

| Author, Year, Country | Study aim type | Study Design | Sample size (N) | Alert Mechanism Type | Device Type |
| --- | --- | --- | --- | --- | --- |
| Becking-Verhaar et al., (2023), Netherlands, (22) | Implementation and Feasibility of Continuous Monitoring Systems | Cross-sectional survey | 58 Nurses | Threshold Alert | Wearable Devices |
| Bellomo et al., (2012), United States, Europe, Australia, (23) | Impact on Clinical Outcomes and Patient Safety | Observational study | 18,305 patients (9,617 before intervention, 8,688 after intervention) | EWS base alerts | Bedside Monitors |
| Blankush et al., (2016), United States, (24) | Technological Evaluation and Alarm Strategies | Observational study | 133 patients | EWS base alerts | Bedside Monitors |
| Brown et al., (2014), United States, (25) | Impact on Clinical Outcomes and Patient Safety | Randomized controlled trial | Baseline cohort: 1535 (control), 1433 (intervention), Post-implementation cohort: 2361 (control), 2314 (intervention) | Threshold Alerts | Bedside Monitors |
| Downey et al., (2018 a), United Kingdom, (26) | Nurses’ and Patients’ Perspectives and Experiences | Randomized controlled trial | 226 patients randomized (140 to continuous monitoring, 86 to intermittent monitoring) | Threshold Alerts | Wearable Devices |
| Downey et al., (2018 b), United Kingdom, (27) | Comparison with Episodic Monitoring | Observational study | 12 patients | Threshold Alerts | Wearable Devices |
| Downey et al., (2020), United Kingdom, (28) | Implementation and Feasibility of Continuous Monitoring Systems | Randomized controlled trial | 136 patients | Threshold Alerts | Wearable Devices |
| Eddahchouri et al., (2022), Netherlands, (29) | Comparison with Episodic Monitoring | Observational study | Baseline cohort: 2466 admissions/ Intervention cohort: 2303 admissions | Threshold Alerts | Wearable Devices |
| Gazarian et al., (2014), United States, (30) | Nurses’ and Patients’ Perspectives and Experiences | Prospective, descriptive, observational study | 57 patients observed, 37 on continuous ECG monitoring. 9 Nurses | Threshold Alerts | Wearable Devices |
| Hravnak et al., (2011), United States, (31) | Technological Evaluation and Alarm Strategies | Observational study | 629 patients (323 in Phase I, 306 in Phase III) | AI-based alerts | Bedside Monitors |
| Hravnak et al., (2008), United States, (32) | Technological Evaluation and Alarm Strategies | Observational study | 326 patients | AI-based alerts | Bedside Monitors |
| Joshi et al., (2022), United Kingdom, (33) | Technological Evaluation and Alarm Strategies | Observational study | 50 patients | Threshold alerts | Wearable Devices |
| Klumpner et al., (2018), United States, (34) | Technological Evaluation and Alarm Strategies | Observational study | 64 monitored rooms. | EWS base alerts | Bedside monitor + Wearable Devices |
| Kuznetsova et.al, (2023), United States, (35) | Implementation and Feasibility of Continuous Monitoring Systems | Observational study | 35 (Pre-implementation: 13, Post-implementation: 22) clinicians | Threshold alerts | Bedside Monitors |
| J. P. L. Leenen et al., (2022), Netherlands, (36) | Implementation and Feasibility of Continuous Monitoring Systems | Observational study | 12 Nurses | Threshold Alerts | Wearable Devices |
| Leenen et al., (2021), Netherlands, (37) | Implementation and Feasibility of Continuous Monitoring Systems | Observational study | 30 patients, 23 Nurses | Threshold Alerts | Wearable Devices |
| McGrath et al., (2019), United States, (38) | Impact on Clinical Outcomes and Patient Safety | Observational study | Two surgical units, 71 beds total | Threshold alerts | Wearable Devices |
| McGrath et al., (2016), United States, (39) | Impact on Clinical Outcomes and Patient Safety | Observational study | Pre-implementation: 4324 patient days post-implementation: 4382 patient days | Threshold Alerts | Wearable Devices |
| McGrath, Taenzer, et al., (2016), United States, (40) | Technological Evaluation and Alarm Strategies | Observational study | General care units, including a 36-bed orthopaedics unit and other surgical and medicine units, Not specified in terms of exact numbers for participants, but system covered more than 200 inpatient beds | Threshold Alerts | Wearable Devices |
| Mestrom et al., (2019), Netherlands, (41) | Impact on Clinical Outcomes and Patient Safety | Observational study | Control group: 320 patients, Intervention group: 274 patients | EWS base alerts | Bedside monitor |
| Paul et al., (2019), Canada, (42) | Impact on Clinical Outcomes and Patient Safety | Randomized controlled trial | Control group: 126 patients, Intervention group: 124 patients | Threshold alerts | Wearable Devices |
| Peelen et al., (2023), Netherlands, (43) | Nurses’ and Patients’ Perspectives and Experiences | Observational study | 1529 patients | AI-based alerts | Wearable Devices |
| Pollack et al., (2009), United States, (44) | Implementation and Feasibility of Continuous Monitoring Systems | Observational study | 298 patients | Threshold alerts | Wearable Devices |
| Posthuma et al., (2023),  Netherlands (45) | Implementation and Feasibility of Continuous Monitoring Systems | Observational study | 742 patients (515 intermittent monitoring, 227 continuous monitoring) | Threshold alerts | Wearable Devices |
| Sigvardt et al., (2024), Denmark, (46) | Nurses’ and Patients’ Perspectives and Experiences | Observational study | 20 patients | Threshold Alerts | Wearable Devices |
| Stellpflug et al., (2021), United States, (47) | Impact on Clinical Outcomes and Patient Safety | Observational before-and-after study | 547 patients during the intervention period, 27 Nurses | Threshold Alerts | Wearable Devices |
| Subbe et al., (2017), United Kingdom, (48) | Impact on Clinical Outcomes and Patient Safety | Observational before-and-after study | Control: 2139 patients, Intervention: 2263 patients | Threshold alert | Bedside Monitors + Wearable Devices |
| Taenzer et al., (2010), United States, (49) | Impact on Clinical Outcomes and Patient Safety | Observational before-and-after study | Pre-implementation: 3118 discharges (intervention unit), 1260 (Comparison unit 1), 2628 (Comparison unit 2), post-implementation: 2841 discharges (intervention unit), 1162 (Comparison unit 1), 2389 (Comparison unit 2), 60 Nurses | Threshold alert | Wearable Devices |
| Un et al., (2021), China, (50) | Implementation and Feasibility of Continuous Monitoring Systems | Observational study | 34 patients | AI-based alerts | Wearable Devices |
| van Goor et al., (2021), Netherlands, (51) | Implementation and Feasibility of Continuous Monitoring Systems | Observational before-and-after study | 209 patients (93 intermittent monitoring, 121 continuous monitoring) | Threshold alert | Wearable Devices |
| van Rossum et al., (2021), Netherlands, (52) | Comparison with Episodic Monitoring | Observational retrospective study | 39 patients | Threshold Alerts | Wearable Devices |
| Verrillo et al., (2019), United States, (53) | Comparison with Episodic Monitoring | Observational study | Preintervention: 427 patients, Intervention: 422 patients | Threshold Alerts | Wearable Devices |
| Watkins et al., (2016), United States, (54) | Impact on Clinical Outcomes and Patient Safety | Prospective observational study | 236 patients, 24 Nurses | AI-based alerts | Wearable Devices |
| Weenk et al., (2019), Netherlands, (55) | Comparison with Episodic Monitoring | Randomized controlled trial | 60 patients | EWS base alerts | Wearable Devices |
| Weller et al., (2018), United States, (56) | Impact on Clinical Outcomes and Patient Safety | Prospective, observational study | 736 patients, 23 nurses and 20 nursing assistants | Threshold Alert | Wearable Devices |

**Table 2 (2)**

| Author, Year | Effectiveness | Efficiency | Satisfaction | Usability Barrier Factors |
| --- | --- | --- | --- | --- |
| Becking-Verhaar et al.,(2023) | N/A | Workload Impact: Nurses noted that continuous monitoring allows for quicker loading of vital signs into EHR compared to manual input.  Time Saving: Saved time primarily in evening and night shifts | Acceptability: High agreement; 97% positive about continuous monitoring. Enhanced patient safety and workflow efficiency,  Comfortability: Mixed feedback; device comfort issues noted, some discomfort due to device size and cables  Qualitative Data: Positive impact on patient care by reducing disturbances. | Issues with device connectivity, battery life, and sensor attachment, Nurses' knowledge and training needs, Internet connectivity issues affecting device performance, Device design and technical concerns affecting usability |
| Bellomo et al., (2012) | RRT calls: Increase in proportion of calls triggered by respiratory criteria (from 21% to 31%; p = .029)  Mortality rate: Increased survival to hospital discharge or 90 days for RRT call patients (from 86% to 92%; p = .04)  Hospital stay length: Decreased in U.S. patients (from 3.4 to 3.0 days; p < .0001) | Workload Impact: Reduced workload required to measure and record vital signs from 4.1 ± 1.3 mins to 2.5 ± 0.5 mins (p < .0001)  Time Saving: Time required to measure and record vital signs decreased significantly | N/A | N/A |
| Blankush et al., (2016) | N/A | Alarm Frequency (per day/ hour): Total alarms: 3.3 per hour, Abnormal vital signs alarms: 2.0 per hour (70% false alarms), Pages for desaturation: 0.1 per hour (55% false alarms), Elevated MEWS notifications: 0.1 per hour (62% false scores),  False Alert rate: 70% for abnormal vital signs,  Workload Impact: Increased due to false alarms, | N/A | Frequent false alarms, patient discomfort with the device. (Discussion only) |
| Brown et al., (2014) | ICU Transfer Rate: No significant change (26.52 vs. 25.93 per 1000 patients, P = 0.92)  Hospital Stay Length: Significantly lower in the intervention unit post-implementation (3.63 days) compared to pre-implementation (4.00 days) and control unit post-implementation (3.61 days) (P < .01) | N/A | N/A | N/A |
| Downey et al., (2018 a) | Mortality rate: One inpatient death due to alcoholic liver disease in the continuous monitoring group  Hospital stay length: Shorter average length of stay in the continuous monitoring group (13.3 days vs 14.6 days)  Readmission: Lower rate of readmission within 30 days in the continuous monitoring group (11.4% vs 20.9%) | N/A | Comfortability: 82% of patients in the continuous monitoring group found the patch comfortable.  Acceptability: 82% of patients in the continuous monitoring group reported feeling safer while wearing the patch. | High rate of initial alerts  Discomfort and itchiness from the patch  Need for patient education on device use and expectations |
| Downey et al., (2018 b) | N/A | N/A | Comfortability: Most patients (10 out of 12) found the patch comfortable and often forgot they were wearing it. One patient found the patch uncomfortable due to feeling heavy after a while.  Acceptability: Most patients (11 out of 12) felt safer wearing the continuous monitoring device. Some patients expressed concerns about the reliability of the technology and the potential loss of personal interaction with nurses. | Potential reduction in face-to-face nursing contact  Some discomfort and concerns about practicalities (e.g., showering with the patch)  Trust issues related to technology reliability and data security |
| Downey et al., (2020) | ICU transfer rate: Fewer unplanned critical care admissions in the continuous monitoring group (1 versus 5).  Mortality rate: One inpatient death in the continuous monitoring group.  Hospital stay length: Shorter average length of hospital stay in the continuous monitoring group (11.6 days vs 16.2 days).  Readmission: Lower rate of readmission within 30 days in the continuous monitoring group (10.2% vs 7.7%). | N/A | Comfortability: Most patients found the patch comfortable (7 out of 52 participants discontinued due to discomfort).  Acceptability: High acceptability; most participants felt safer wearing the patch. | Patient discomfort and skin reactions.  Too many false alerts leading to inconvenience.  (Discussion only) |
| Eddahchouri et al., (2022) | RRT Calls: Decreased from 107 (4.3%) to 71 (3.1%), P=0.02  ICU Transfer Rate: Decreased from 84 (3.4%) to 54 (2.3%), P=0.03  Mortality Rate: No significant difference  Hospital Stay Length: No significant difference  Other: Rapid response team calls that did not result in ICU admission decreased (70 [2.8%] vs. 45 [2.0%], P=0.05). Rapid response team calls that resulted in ICU admission were not significantly different (52 [2.1%] vs. 36 [1.6%], P=0.16). | N/A | N/A | N/A |
| Gazarian et al., (2014) | N/A | Alarm Frequency (per day/ hour): 3.79 alarms per monitored hour (total 205 alarm events during 54 hours of observation)  False Alert rate: High false alarm rate, especially for crisis level alarms (e.g., all 17 crisis level alarms were false alarms).  Workload Impact: Nurses responded to 46.8% of alarms. High rate of false alarms leading to alarm fatigue and potential desensitization. | N/A | High false alarm rate leading to alarm fatigue.  Inconsistent practices in checking and managing alarm parameters.  Lack of designated monitor watchers, increasing the burden on nurses. |
| Hravnak et al., (2011) | ICU transfer rate: Decreased in Phase III (not quantified in terms of percentage)  Mortality rate: Decreased unexpected deaths in Phase III  Serious adverse events: Reduction in INSTABILITYfull episodes (serious instability) | False Alert rate: INDEXmin correlated with INSTABILITYmin in 43% of cases. INSTABILITYmin episodes not accompanied by INDEX alert were short in duration (98% lasted <4 minutes).  Workload Impact: Reduced cognitive burden due to fewer false alarms and early detection of instability. | N/A | N/A |
| Hravnak et al., (2008) | ICU transfer rate: Decreased in Phase III (not quantified in terms of percentage)  Mortality rate: Decreased unexpected deaths in Phase III  Serious adverse events: Reduction in INSTABILITYfull episodes (serious instability) | False Alert rate: INDEXmin correlated with INSTABILITYmin in 43% of cases. INSTABILITYmin episodes not accompanied by INDEX alert were short in duration (98% lasted <4 minutes).  Workload Impact: Reduced cognitive burden due to fewer false alarms and early detection of instability. | N/A | N/A |
| Joshi et al., (2022) | Other: 2 patients initiated on the sepsis pathway. 3 patients referred to a senior clinician. Average time to staff acknowledgment of alerts was 154 minutes. | Other: Nurses acknowledged 27 out of 51 alerts. | N/A | N/A |
| Klumpner et al., (2018) | N/A | Alarm Frequency (per day/ hour): Automated system generated one page every 2.3 hours.  False Alert rate: By liberalizing the MEWC criteria, the system reduced the potential frequency of pages to an acceptable level.  Workload Impact: Reduced cognitive burden on clinical staff by minimizing unnecessary pages.  Time Saving: Anecdotally noted to improve timely recognition of patient deterioration. | N/A | N/A |
| Kuznetsova et.al, (2023) | N/A | Alarm Frequency (per day/ hour): One minute and four seconds on average response time  Workload Impact: Less alarm fatigue than anticipated, improved trust in CFCM  Time Saving: Immediate response times below two-minute target | Acceptability: Nurses reported high satisfaction with the training  Other: Concerns about impact on patient experience, such as alarm fatigue for patients | Pre-implementation barriers included alarm fatigue, accuracy and trust issues, impact on patient experience, and challenges to status.  Post-implementation challenges included insufficient training for secondary users, impact on patient experience |
| J. P. L. Leenen et al., (2022) | Nurses reported feeling that continuous monitoring could improve patient safety by providing better insight into vital sign trends and potentially detecting clinical deterioration earlier. | Alarm Frequency (per day/hour): Nurses reported the number of alarms and false alarms as excessive, leading to irritation and uncertainty.  False Alert Rate: High false alarm rate due to system's strict artifact rejection algorithms.  Workload Impact: Nurses found the quantity of alarms disruptive, and it caused additional workload. | Other: Nurses expressed a preference for integration of the monitoring system into existing mobile devices and EMR for better usability.  Nurses hoped that in the future, wearable sensors could reduce the need for manual vital sign measurements, thus saving time.  Some nurses suggested user-adjustable alarm settings to reduce false alarms and prevent alarm fatigue. | Management of alarms: Excessive (false) alarms generated by the system.  Integration with clinical workflow: Challenges with managing clinical workflows and ensuring seamless integration with existing hospital systems.  Learning and coaching: Need for ongoing training and coaching to build confidence and competence in using the system.  Interpreting vital sign trends: Difficulty in interpreting trends and determining appropriate follow-up actions. |
| Leenen et al., (2021) | Hospital stay length: Median length of stay: 4.0 (IQR 3.75–13.0) days  Other: 36.7% of patients developed complications. | Alarm Frequency (per day/hour): Median alert rate of 4.5 per patient per day  False Alert Rate: 44% of vital sign alerts were false positives | Comfortability: 93% of patients rated the patch as comfortable  Acceptability: 89% of patients would like to wear it next time in the hospital | N/A |
| McGrath et al., (2019) | N/A | Alarm Frequency (per day/hour): No significant increase in clinical alarms per monitored hour.  Workload Impact: Decrease in the time required to perform vital signs assessment, allowing staff to spend more time on additional patient-focused tasks.  Time Saving: Significant decrease in time required to obtain and record vital signs (mean assessment times were 178.8 sec before vs. 128.9 sec after implementation). | Acceptability: High staff satisfaction ratings  Other: 79% of LNAs and 78% of RNs rated overall system satisfaction 4 or 5 on a 5-point scale. 94% of RNs and 97% of LNAs cited 100% use of the system with their patients during the study period. | N/A |
| McGrath et al., (2016) | N/A | Alarm Frequency (per day/hour): Median alarm rate was approximately one per patient per nursing shift.  Workload Impact: No significant workflow issues reported; moderate patient acceptance. | Acceptability: Moderate acceptance rate (57% utilization). | Patient refusal due to sensor discomfort (22.7%)  Restriction of movement (3.4%)  Technical difficulties (2.9%)  Patient confusion (2.7%)  Skin problems (2.2%) |
| McGrath, Taenzer, et al., (2016) | RRT Calls: Reduction of rescue events by more than 60% in 2008  ICU Transfer Rate: Reduction of unplanned transfers by 50% in 2008 | Alarm Frequency (per day/hour): Approximately 2 alarms per patient per 12-hour nursing shift in the pilot unit  Workload Impact: Significant reduction in alarm exposure for nurses | Acceptability: High level of staff adoption and preference for the new system over the previous system with high false alarm rates | Management of alarms: High false alarm rates with previous systems; improved with new system.  Integration with clinical workflow: System designed to integrate with nursing workflow and reduce disruptions.  (Discussion only) |
| Mestrom et al., (2019) | Mortality rate: No significant difference  Hospital stay length: No significant difference  Readmission: No significant difference  Other: Improved adherence to MEWS protocol, increased complete MEWS assessments (1.1% to 25.4%) | N/A | N/A | N/A |
| Paul et al., (2019) | ICU transfer rate: 1 event in the wireless group, none in the control group | Alarm Frequency (per day/hour): 4.0 alarms per week in the wireless group | Comfortability: High tolerance rate (86.6% completion rate)  Acceptability: High acceptance rate among patients | Baseline tachycardia causing multiple alarms  Multiple false alarms due to malfunctioning hardware  Patient refusal due to discomfort or personal reasons  (Discussion only) |
| Peelen et al., (2023) | Other: Continuous VSI warned for 55% of escalation of care vs. 51% by periodic EWS. Continuous monitoring warned earlier: 8.3 hours (IQR: 2.6-24.8) vs. 5.2 hours (IQR: 2.7-12.3) with EWS (P=0.074). | Alarm Frequency (per day/hour): 0.99 warnings per day per patient with continuous monitoring vs. 0.13 with periodic EWS.  False Alert rate: Higher for continuous monitoring compared to periodic EWS. | N/A | Monitoring was discontinued in some patients due to reasons like delirium, contact allergies, initiation of palliative care, or late opting out of monitoring.  (Discussion only) |
| Pollack et al.,(2009) | Other: Productive clinical alarms (those that prompted a change in patient therapy, location, or intensity of monitoring) occurred in 20 patients (6.7%). | False Alert rate: 10 false-positive alarms in 4 patients (1.3%).  Time Saving: Prompted earlier transition of patients from the waiting room to clinical space in the ED. | Comfortability: Patients reported good satisfaction with the device (median scores for various comfort metrics were above 4 out of 5).  Acceptability: Clinician satisfaction was high, with median scores indicating minimal interference with routine care and ease of device application and removal. | Technical and artifactual issues, such as poor lead adherence and interference from patient movements or electronics. |
| Posthuma et al., (2023) | ICU transfer: Twenty-two patients (4.3%) in the control group and 13 patients (5.7%) in the intervention group were admitted to the ICU after an initial postoperative stay on the ward.  Hospital Stay Length: Median length of hospital stay was 5.9 days (Interquartile Range (IQR): 6.4 days) in the control group and 6.0 days (IQR: 8.0 days) in the intervention group.  Others:. A 4.6% reduction in new disability at 3 months was observed in the intervention group compared to control. ICU stay: The median length of stay in the ICU after an initial ward stay was 3.4 days (IQR: 11.0 days) and 6.0 days (IQR: 8.6 days) in the control and intervention group, respectively. | N/A | N/A | N/A |
| Sigvardt et al., (2024) | N/A | Workload Impact: Continuous monitoring reduced the workload significantly.  Time Saving: Median difference of 9.9 minutes per patient per day (p < .001). The time used for continuous monitoring was 6.0 minutes per patient per day vs. 14 minutes per patient per day for NEWS. | N/A | N/A |
| Stellpflug et al., (2021) | RRT calls: Decreased by 53% from 55 (baseline) to 26 (intervention period)  ICU transfer rate: Decreased from 26 (2016) and 28 (2017) to 17 (2018), but not significantly  Hospital stay length: ICU LOS decreased from 2.82 days (baseline) to 2.19 days (intervention) | Workload Impact: Nurses reported the device was time-consuming initially but improved over time. The monitor was integrated into nurses' workflow effectively, leading to better decision-making.  Time Saving: Nurses had more time for other patient care tasks as proficiency with the device increased. | Comfortability: Patients found the device comfortable to wear.  Acceptability: 74% of nurses reported the device enhanced decision-making; patients felt safer and appreciated not being disconnected from a wall monitor. | Initial time-consuming and cumbersome nature of the device  Removal and reapplication of the device for showers or tests were time-consuming |
| Subbe et al., (2017) | RRT calls: Increased from 405 to 524 (p = 0.001)  ICU transfer rate: ICU admissions reduced, but specific rate not detailed  Mortality rate: Decreased from 173 to 147 (p = 0.042)  Serious adverse events: Significant reduction in serious events (p < 0.001) | N/A | N/A | N/A |
| Taenzer et al., (2010) | RRT calls: Rescue events decreased from 3.4 (1.89–4.85) to 1.2 (0.53–1.88) per 1000 patient discharges in the intervention unit.  ICU Transfer Rate: Decreased from 5.6 (3.7–7.4) to 2.9 (1.4–4.3) per 1000 patient days in the PSS unit  Mortality Rate: Decreased from 4 to 2 deaths post-implementation  Hospital Stay Length: No significant change, pre-implementation: 3.69 days, post-implementation: 3.68 days | Alarm Frequency (per day/hour): 4 alarms per patient per day or two per 12-hour nursing shift  False Alert Rate: Thoughtful implementation and careful calibration of alarm triggers minimized false alarms and ensured clinical acceptance. | Acceptability: High level of staff acceptance and preference for the new system due to fewer nuisance alarms and more actionable alerts. The system had a very high patient acceptance rate of 98.2%. | Management of alarms: High false alarm rates with previous systems; improved with new system.  Integration with clinical workflow: Challenges with managing clinical workflows and ensuring seamless integration with existing hospital systems. |
| Un et al., (2021) | Serious Adverse Events: 17 clinical worsening events were detected in 34 patients.  Hospital Stay Length: Median hospital stay was 15 days. | False Alert Rate: 2 false positive alerts without actual clinical worsening events within the subsequent 3 days.  Workload Impact: Reduced manual measurements, thus reducing healthcare workers' exposure to COVID-19 and conserving PPE. | N/A | N/A |
| van Goor et al., (2021) | N/A | Workload Impact: Increased the total number of used personal protection equipment per room per shift from 15 (IQR 8.5–22) to 19 (IQR 12–25). Total room visits did not significantly change after introducing continuous monitoring. MEWS visits decreased over time, but this decline was not influenced by the intervention. Evening and night shifts had fewer patient room visits compared to day shifts. | N/A | N/A |
| van Rossum et al., (2021) | Serious Adverse Events: 18 adverse events detected in 14 patients | Alarm Frequency (per day/hour): 0.49 alarms per patient per day  False Alert Rate: 59% of alarms classified as false positive | N/A | N/A |
| Verrillo et al., (2019) | RRT calls: No significant change.  ICU transfer rate: Decrease from 8.5 to 7.2 per 1000 patient days (not statistically significant).  Mortality rate: Decrease to zero in the intervention group (not statistically significant).  Serious adverse events: Decrease in the complication rate from 22% to 5.9%. | N/A | Acceptability: Positive, with nurses preferring cVSM over eVSC. | N/A |
| Watkins et al., (2016) | Other: 92% of nurses agreed that the number of alarms and alerts were appropriate; 54% strongly agreed. 100% of nurses agreed that the monitor provided valuable patient data that increased patient safety; 79% strongly agreed. | Alarm Frequency (per day/hour): On average, 10.8 alarms per patient per day.  Workload Impact: Nurses reported that the number of alarms and alerts were appropriate. | N/A | N/A |
| Weenk et al., (2019) | Continuous monitoring detected 71 high MEWS periods with ViSi Mobile and 32 high MEWS periods with HealthPatch during non-observed periods by nurses. | Continuous monitoring may provide earlier detection of clinical deterioration, potentially improving timeliness of clinical actions. | N/A | N/A |
| Weller et al., (2018) | RRT calls: Decrease from 189 to 158 per 1000 discharges (p < 0.05)  ICU transfer rate: Decrease from 53 to 40 per 1000 discharges (not statistically significant)  Mortality rate: Decrease (not statistically significant)  Hospital stay length: No significant difference | Alarm Frequency: 2.3 alarms per patient per day  False Alert rate: initially found an alarm rate of over 11 alarms per patient per day. Through adjustments, the final alarm rate was reduced to 2.01 alarms per patient per day.  Workload Impact: Acceptable to nursing staff  Time Saving: Implied by reduced alarm frequency and intervention times | Acceptability: High among nursing staff | N/A |

**Table 2(1) and Table (2) are together, but it is too big so break it apart.**
