# Supplementary material for: The Usability of Continuous Monitoring Devices With Deterioration Alerting Systems in Noncritical Care Units: Scoping Review
Source: Interact J Med Res. 2026 Feb 10;15:e75713. doi: 10.2196/75713 (PMC12892175; doi:10.2196/75713)
Supplement: Multimedia Appendix 4 [file ijmr-v15-e75713-s004.docx]

**Attachment 3 Characteristics of Included studies**

| Categories | Number of studies (%) | Reference |  |
| --- | --- | --- | --- |
| Aim type of the study (n=35) |  |  |  |
| Implementation and Feasibility of Continuous Monitoring Systems^a^  Comparison with Episodic Monitoring^b^  Impact on Clinical Outcomes and Patient Safety^c^  Nurses’ and Patients’ Perspectives and Experiences^d^  Technological Evaluation and Alarm Strategies^e^ | 9 (26)  5 (14)  11 (31)  4 (11)  6 (17) | (22, 28, 35-37, 44, 45, 50, 51)  (27, 29, 52, 53, 55)  (23, 25, 38, 39, 41, 42, 47-49, 54, 56)  (26, 30, 43, 46)  (24, 31-34, 40) |  |
| 1. Implementation and Feasibility of Continuous Monitoring Systems: Studies that explore the practical challenges, acceptance, and operational integration of continuous monitoring systems in clinical settings. 2. Comparison with Episodic Monitoring: Studies that evaluate the differences in effectiveness, response times, and clinical outcomes between continuous and episodic (intermittent) vital sign monitoring methods. 3. Impact on Clinical Outcomes and Patient Safety: Studies that assess the effects of continuous monitoring on patient outcomes, safety measures, and the prevention of adverse events. 4. Nurses’ and Patients’ Perspectives and Experiences: Studies that investigate the experiences, satisfaction, and perceived benefits and challenges of continuous monitoring from the perspectives of nurses and patients. 5. Technological Evaluation and Alarm Strategies: Studies that evaluate the performance, reliability, and optimization of monitoring technologies and alarm strategies to enhance patient care. | | |  |
| Country (n=35) |  |  |  |
| United States  Netherlands  United Kingdom  United States, Europe, Australia  Others | 16 (46)  9 (29)  5 (14)  1 (3)  3 (9) | (24, 25, 30-32, 34, 35, 38-40, 44, 47, 49, 53, 54, 56)  (22, 29, 36, 37, 41, 43, 45, 51, 52, 55)  (26-28, 33, 48)  (23)  (42, 46, 50) |  |
| Study Design (n=35) |  |  |  |
| Observational study (prospective, retrospective before-and-after)  Randomized control trial  Cross-sectional survey | 28 (71)  6 (12)  1 (3) | (23, 24, 27, 29-41, 43, 44, 46-54, 56)Put in  (25, 26, 28, 42, 45, 55)  (22) |  |
| Study Design type (n=35) |  |  |  |
| Qualitative  Randomized control trial  Non-Randomized  Quantitative Descriptive  Mixed Method | 3 (9)  5 (14)  20 (57)  5 (14)  2 (6) | (27, 35, 36)  (26, 28, 42, 45, 55)  (23-25, 29, 31-33, 38, 39, 41, 43, 44, 47-52, 54, 56)  (30, 34, 37, 46, 53)  (22, 40) |  |
| Comparison Group (n=35) |  |  |  |
| Before-and-after implementation Comparison  Comparisons with intermittent monitoring  Comparisons with baseline data  Comparison between monitor or alert method  No comparison | 11 (31)  12 (34)  1 (3)  2 (6)  9 (26) | (23-25, 31, 32, 35, 39, 40, 49, 51, 56)  (26, 28, 29, 34, 38, 41-43, 45, 46, 48, 53)  (47)  (52, 55)  (22, 27, 30, 33, 36, 37, 44, 50, 54) |  |
| Data Collection Method (n=35) |  |  |  |
| Methods without survey or interview (Electronic Health Record, Observation or Clinical Trials and Experiments)  Methods involve Survey  Methods involve interview  Methods involve Survey and Interview | 22 (63)  10 (29)  2 (6)  1 (3) | (23-25, 29-34, 39, 41-43, 45, 46, 48-53, 56)  (22, 26, 28, 37, 38, 40, 44, 47, 54, 55)  (27, 35)  (36) |  |
| Clinical settings in the study (n=35) |  |  |  |
| Surgical Related Wards  General Wards  Surgical and Internal Unit  Others | 18 (51)  7 (20)  1 (3)  9 (26) | (22, 24-27, 29, 30, 35, 36, 38-40, 42, 43, 45, 49, 52, 54)  (23, 37, 46-48, 51, 53)  (55)  (28, 31-34, 41, 44, 50, 56) |  |
| Patient Type (n=35) | |  |  |
| Postoperative  General medical, trauma, and surgical patients  Respiratory include (COVID-19)  Other  Not Specify | 17 (49)  1 (3)  4 (11)  6 (17)  7 (20) | (26-30, 33, 37-39, 41-43, 45, 49, 52-54)  (25)  (24, 48, 50, 51)  (31, 32, 34, 46, 47, 56)  (22, 23, 35, 36, 40, 44, 55) |  |
| Devices Name (n=35) | |  | |
| SensiumVitals patch^a^  ViSi Mobile^b^  Patient SafetyNet^c^  Philips IntelliVue Guardian Solution^d^  Other | 8 (23)  7 (20)  4 (11)  4 (11)  12 (34) | (26-28, 33, 36, 37, 45, 52)  (22, 43, 51, 53-56)  (38-40, 49)  (23, 24, 41, 48)  (25, 29-32, 34, 35, 42, 44, 46, 47, 50) | |
| 1. SensiumVitals patch: It, manufactured by Sensium Healthcare, is a wearable device applied to the chest that continuously monitors HR, RR, and BT. It transmits data wirelessly every two minutes to a central monitoring station or a mobile device, alerting healthcare providers of any deviations from preset physiological norms with visual alerts and reminders. 2. ViSi Mobile: It, manufactured by Sotera Wireless, is a wearable device that monitors HR, BP, RR, BT and SpO2 from the upper arm, chest, and wrist. It transmits visual alerts to a central monitor and the nurse's Wi-Fi phone, ensuring continuous patient monitoring. 3. Patient SafetyNet: It, manufactured by Masimo, is a remote monitoring and clinician notification system that displays near real-time data from connected Masimo or third-party devices at a central station. It monitors vital signs such as HR and SpO2, sending visual alerts and audible alarms directly to clinicians, with escalations to additional staff if alarms are not acknowledged promptly. Finger, toe, and ear probes 4. Philips IntelliVue Guardian Solution: It, manufactured by Philips, is a bedside monitoring system that tracks vital signs including HR, RR, BP, BT, and SpO2. It provides visual alerts on central and bedside monitors and can be used with wireless monitors applied to the chest, wrist, and upper arm, ensuring continuous patient monitoring and timely clinical actions. | | | |
| Devices Type (n=35) |  |  | |
| Wearable devices  Bedside monitors  Bedside Monitors + Wearable Devices | 26 (74)  5 (14)  4 (11) | (22, 26-30, 33, 36-40, 42-47, 49-56)  (23-25, 35, 41)  (31, 32, 34, 48) | |
| Alert Path (n=35) |  |  | |
| Alerts at central stations or system  Alert to central station or system and clinicians' phones or mobile devices | 14 (40)  21 (60) | (22, 23, 29-32, 35, 38, 41, 43, 44, 47, 50, 54)  (24-28, 33, 34, 36, 37, 39, 40, 42, 45, 46, 48, 49, 51-53, 55, 56) | |
| Alert Mechanism Type (n=35) | |  | |
| Threshold alerts  Early warning score-based alerts  Artificial intelligence-based alerts | 25 (71)  5 (14)  5 (14) | (22, 25-30, 33, 35-40, 42, 44-49, 51-53, 56)  (23, 24, 34, 41, 55)  (31, 32, 43, 50, 54) | |
