# Supplementary material for: The Usability of Continuous Monitoring Devices With Deterioration Alerting Systems in Noncritical Care Units: Scoping Review
Source: Interact J Med Res. 2026 Feb 10;15:e75713. doi: 10.2196/75713 (PMC12892175; doi:10.2196/75713)
Supplement: Multimedia Appendix 5 — Summary of the clinicians’ characteristics in the included studies. [file ijmr-v15-e75713-s005.docx]

**Attachment 4 Summary of clinicians’ characteristics in included studies**

| Studies (n=10) | Sample size (N) | Male %: Female % ratio, mean age (years) | Clinician Experience |
| --- | --- | --- | --- |
| Becking-Verhaar et al., (2023) | **58** | **15.5% Male, 84.5% Female, not specified** | **Not specified** |
| Gazarian et al., (2014) | **9** | **Males: 0%, Females: 100%, Mean age: 29.75 years (range 23-45)** | **Average of 6 years (range 1-25) of work experience** |
| Kuznetsova et al., (2023) | **35 (Pre-implementation: 13, post-implementation: 22)** | **5.7% Male, 94.3% Female** | **9.3 years** |
| J. P. L. Leenen et al., (2022) | **12** | **All female, median age: 27.5 years (IQR 23–31.5)** | **Median of 5.5 years (IQR 2–8.5) of work experience** |
| Jobbe P. L. Leenen et al., (2021) | **23** | **Not specified; Median age: 28 years** | **Median working experience: 5 years** |
| Stellpflug et al., (2021) | **27** | **Not specified** | **Not specified** |
| Taenzer et al., (2010) | **60** | **Not specified** | **Not specified** |
| Verrillo et al., (2019) | **First survey: 17 out of 34, Second survey: 15 out of 29** | **All female, not specified** | **Mean length of employment: 7.7 years (range: 7 months to 33.3 years).** |
| Watkins et al., (2016) | **24** | **Not specified** | **Not specified** |
| Weller et al., (2018) | **23 nurses and 20 nursing assistants** | **Not specified** | **Not specified** |
